# Expert consensus on antimicrobial resistance research priorities to focus development and implementation of antibacterial vaccines and monoclonal antibodies

**DOI:** 10.2807/1560-7917.ES.2024.29.47.2400212

**Published:** 2024-11-21

**Authors:** Nasreen Hassoun-Kheir, Mariana Guedes, Fabiana Arieti, Maria Diletta Pezzani, Beryl Primrose Gladstone, Julie V Robotham, Koen B Pouwels, Rhys Kingston, Yehuda Carmeli, Alessandro Cassini, Michele Cecchini, Francis Drobniewski, Isabel Frost, Jeroen Geurtsen, Andreas Kronenberg, Mila Nu Nu Htay, Mical Paul, Nuno Rocha-Pereira, Jesús Rodríguez-Baño, Luigia Scudeller, Andrew J Stewardson, Evelina Tacconelli, Stephan Harbarth, Venanzio Vella, Marlieke EA de Kraker

**Affiliations:** 1 Infection Control Program, Geneva University Hospitals and Faculty of Medicine, WHO Collaborating Center, Geneva, Switzerland; 2 Instituto de Biomedicina de Sevilla (IBiS), Infectious Diseases and Microbiology Division, Hospital Universitario Virgen Macarena, Department of Medicine, University of Sevilla/CSIC, Sevilla, Spain; 3 Infection and Antimicrobial Resistance Control and Prevention Unit, Hospital Epidemiology Centre, Centro Hospitalar Universitário São João, Porto, Portugal; 4 Infectious Diseases, Department of Diagnostics and Public Health, University of Verona, Verona, Italy; 5 DZIF-Clinical Research Unit, Infectious Diseases, Department of Internal Medicine, University Hospital Tübingen, Tübingen, Germany; 6 HCAI, Fungal, AMR, AMU & Sepsis Division, UK Health Security Agency, London, United Kingdom; 7 Health Economics Research Centre, Nuffield Department of Population Health, University of Oxford, Oxford, United Kingdom; 8 National Institute for Antibiotic Resistance and Infection Control, Ministry of Health, Tel Aviv; Faculty of Medicine, Tel Aviv University, Tel Aviv, Israel; 9 Infectious Diseases Service, Lausanne University Hospital, Lausanne, Switzerland and Public Health Department, Canton of Vaud, Lausanne, Switzerland; 10 Head of Public Health, Health Division, OECD (Organisation for Economic Co-operation and Development), Paris, France; 11 Department of Infectious Disease, Imperial College London, London, United Kingdom; 12 AstraZeneca, Eastbrook House, Cambridge, United Kingdom; 13 Bacterial Vaccines Research & Early Development, Janssen Vaccines & Prevention B.V., Leiden, the Netherlands; 14 Swiss Centre for Antibiotic Resistance, Institute for Infectious Diseases, University of Bern, Bern, Switzerland; 15 Department of Community Medicine, Faculty of Medicine, Manipal University College Malaysia, Melaka, Malaysia; 16 Infectious Diseases Institute, Rambam Health Care Campus; Faculty of Medicine, Technion – Israel Institute of Technology, Haifa, Israel; 17 Department of Medicine, Faculdade de Medicina da Universidade do Porto, Porto, Portugal; 18 CIBERINFEC, Instituto de Salud Carlos III, Madrid, Spain; 19 Research and Innovation Unit, IRCCS Azienda Ospedaliero-Universitaria di Bologna, Bologna, Italy; 20 Department of Infectious Diseases, The Alfred Hospital and School of Translational Medicine, Monash University, Melbourne, Victoria, Australia; 21 GSK, Siena, Italy

**Keywords:** Antimicrobial resistance, Europe, gaps, Delphi, policy, research priority, vaccines, monoclonal antibodies

## Abstract

To reduce antimicrobial resistance (AMR), pathogen-specific AMR burden data are crucial to guide target selection for research and development of vaccines and monoclonal antibodies (mAbs). We identified knowledge gaps through previously conducted systematic reviews, which informed a Delphi expert consultation on future AMR research priorities and harmonisation strategies to support data-driven decision-making. Consensus (≥80% agreement) on importance and feasibility of research topics was achieved in two rounds, involving 24 of 39 and 19 of 24 invited experts, respectively. Priority pathogens and resistance profiles for future research were identified: third generation cephalosporin-resistant *Klebsiella pneumoniae* and *Escherichia coli,* for bloodstream and urinary tract infections, respectively, and meticillin-resistant *Staphylococcus aureus* for surgical-site infections. Prioritised high-risk populations included surgical, haemato-oncological and transplant patients. Mortality and resource use were prioritised as health-economic outcomes. The importance of age-stratified data and inclusion of a non-infected comparator group were highlighted. This agenda provides guidance for future research to fill knowledge gaps and support data-driven selection of target pathogens and populations for new preventive and treatment strategies, specifically vaccines and mAbs, to effectively address the AMR burden in Europe. These research priorities are also relevant to improve the evidence base for future AMR burden estimates.

## Background

The increasing threat of antimicrobial resistance (AMR) and the complexities of developing new antibiotics has stimulated the search for alternative prevention and treatment strategies, such as vaccines and monoclonal antibodies (mAbs) [1,2]. Vaccines can play an important role in preventing and controlling AMR by reducing the incidence of infectious diseases and subsequent antibiotic use and diminishing the emergence of AMR at population level [3]. Following global implementation of pneumococcal conjugate vaccines (PCV), decreased antibiotic resistance was observed in *Streptococcus pneumoniae* isolates [4]. Monoclonal antibodies, on the other hand, can reduce the impact of drug-resistant infections by prophylactic intervention in specific risk groups. They can also be used as a stand-alone/adjuvant treatment once an infection occurs, with much lower risk of resistance development as compared to antibiotic treatments [2]. Bezlotoxumab, a mAb developed for secondary prophylaxis of *Clostridium difficile* was found to significantly reduce the rate of recurrent *C. difficile* infections in clinical trials and in real-world practice [5,6].

Vaccines and mAbs target specific strains, but they do not distinguish between drug-resistant or drug-susceptible pathogens. As such, these types of intervention strategies will not only have the potential to reduce the burden of AMR but will also be able to reduce the overall burden of infections caused by the targeted pathogen. Since the burden of drug-susceptible infections is, in general, larger than the burden of drug-resistant infections, this will have important public health benefits.

In a recently published paper on the vaccine development pipeline, vaccines were categorised as a strategy of moderate or low feasibility for most World Health Organization (WHO) 2017 priority pathogens [7]. Challenges that hamper further development and implementation of potential agents include sufficient agent efficacy, serotype coverage and development costs [2,8,9]. Hence, prioritisation of targets is crucial to guide efficient development of potential agents. Since vaccines, as well as mAbs, are pathogen-specific, burden data broken down to pathogen-level are required to guide target prioritisation in research and development (R&D), and pathogen-infection burden is needed to guide implementation strategies of potential agents.

The public-private partnership project Predicting the Impact of Monoclonal Antibodies & Vaccines on Antimicrobial Resistance (PrIMAVeRa) aims to evaluate the potential cost-effectiveness of vaccines and mAbs with different product profiles to reduce the burden of AMR in Europe. Within the PrIMAVeRa project, modelling approaches are planned to evaluate different implementation scenarios of future vaccine and mAbs and their impact on AMR incidence and associated health-economic outcomes. To inform these models, three systematic reviews were performed focusing on AMR frequency measures [10], excess health risks [11], and additional resource utilisation [12] associated with 22 pathogen-infection combinations in Europe [13]. Although the three systematic reviews identified about 400 relevant studies including specific pathogen-infection combinations, significant knowledge gaps and methodological shortcomings were detected.

In this perspective we report on an exercise to define a future research agenda that could fill key knowledge gaps on AMR burden, stratified by pathogens, infection types and risk groups. This agenda is needed to inform which vaccines and mAbs should be developed to efficiently tackle the burden of AMR, since options to adequately treat drug-resistant infections have become increasingly limited. In addition, we gather recommendations to improve data quality, harmonisation, methodology and reporting of AMR burden estimates.

## Identification of antimicrobial resistance burden knowledge gaps

Availability and quality of data on AMR incidence, excess health risks and additional resource utilisation attributable to, and associated with, AMR were extracted from the three PrIMAVeRa systematic reviews [10-12]. The pathogen selection for the systematic reviews was based on relevance ranking by the United States Centers for Disease Control and Prevention (CDC) [14], the WHO Priority Pathogens list (PPL) [15], European AMR burden estimates [16], the European Centre for Disease Prevention and Control (ECDC) proportions of resistance [17], and the availability of vaccines or mAbs in the clinical development pipeline [7]. As such, the reviews focused on carbapenem-resistant (CR) *Pseudomonas aeruginosa* and *Acinetobacter baumannii*, third generation cephalosporin-resistant (3GCR) or CR *Escherichia coli* and *Klebsiella pneumoniae*, meticillin-resistant *Staphylococcus aureus* (MRSA) and vancomycin-resistant *Enterococcus faecium* (VRE) based on phenotypic resistance. Subsequently, clinically relevant infection types were selected per pathogen during PrIMAVeRa expert meetings, resulting in 22 pathogen-infection combinations appended in Supplementary Table S1. Teams working with the systematic reviews identified data gaps for each of the selected pathogen infection combinations using relevant PICO (patients, intervention/exposure, comparator, outcome) elements (interpretation of each PICO element is highlighted in Supplementary Table S2). The teams then classified the reason for the knowledge gaps as: (i) insufficient or imprecise information, (ii) information at risk of bias, (iii) inconsistent or unknown consistency of information or (iv) the allocated information is not the right information needed to answer the research question [18]. A steering committee of investigators directly involved in the systematic reviews within PrIMAVeRa (NHK, MG, MdK, KP, JR, RK, FA, MDP and BPG) summarised these into problem statements specifying reason(s) for each knowledge gap. This process is detailed in Supplementary Figure S1. General knowledge gaps affecting all four PICO elements for all pathogen-infection combinations were classified as recommendations for future research and were not included as problem statements. For encountered methodological shortcomings related to specific pathogen-infection combinations (reasons ii and iii) problem statements were formulated. Otherwise, general methodological recommendations were issued by the steering committee.

## Obtaining antimicrobial resistance experts’ feedback and consensus via a Delphi process

To achieve consensus on the relevance of all recorded problem statements using Delphi methodology, AMR experts’ feedback was sought in multiple iterative rounds [19] between April and May 2023. Experts were selected based on their background in AMR combined with any of the following: infectious diseases epidemiology, healthcare, public health, health economics or clinical research funding. The selection aimed for balance in gender, age, country of practice (with preference for experts practicing in Europe) and institution (academic, non-governmental organisation, private sector). Candidate experts were also identified through key publications [7,15,16,20,21], proposed by the steering committee and by the PrIMAVeRa consortium. We aimed to obtain responses from at least 20 experts. Experts were not reimbursed for their participation.

A questionnaire with problem statements was drafted in REDCap (Research Electronic Data Capture, hosted by University Hospital of Verona, see Supplementary Material 2, Round 1). Seven external researchers not linked to the PrIMAVeRa consortium piloted the questionnaire to verify clarity of meaning and response time. The selected experts were then invited to participate by email, which included a personal link to the Delphi questionnaire. They were given 2 weeks to respond for each Delphi round, with a reminder sent after 1 week. The first questionnaire asked the experts to assess the importance of filling the knowledge gaps with future research in Europe, considering the reason for the gap. A summary of the existing evidence found in the systematic reviews was provided including the number of studies per pathogen-infection combination per systematic review, as shown in Supplementary Tables S3-S5. Experts were asked to score their agreements with the provided statements based on a Likert scale (1 = strongly disagree, 2 = moderately disagree, 3 = neither agree nor disagree, 4 = moderately agree and 5 = strongly agree, with an additional option of don’t know/no expertise in this field), or to rank options referring to prioritisation of pathogens, infection types, outcomes and risk groups for future research on AMR. When a knowledge gap was due to biased or inconsistent information [18], experts were asked to rank the best future research approaches pertaining to these gaps. Experts were then offered the option to suggest rephrasing, give free text feedback and/or suggest new statements. From the second Delphi round, experts were also asked to assess the feasibility of filling identified knowledge gaps with future research, considering factors such as required study design, sample size, costs, setting and ease of implementation in Europe. All questions are shown in Supplementary Material 2, Round 2. Consensus methods used are provided in detail in the Supplementary Material 1, ‘Consensus approach in Delphi survey’. Aggregated, anonymous feedback was provided to experts in subsequent rounds. Any communication received from the experts on the panel was made available to the other experts indirectly through the steering committee.

## Consensus after two Delphi rounds

On 21 April 2023, 39 experts were invited to participate in the consensus process and 24 responded to the Delphi questionnaire first round. The second Delphi round was launched on 2 May 2023, and 19 of 24 experts responded. Most experts had over 10 years of experience in AMR-related fields and primarily practiced in western or southern European settings. Their areas of expertise included infectious disease epidemiology, health economics, healthcare provision, public health, health policy, global health and clinical AMR research funding. Detailed information on demographics and qualifications of the experts is displayed in Supplementary Table S6.

Only two Delphi rounds were required to reach consensus. The agreement on importance and feasibility of each problem statement is reported in Supplementary Table S7. In the first round, 20 statements were included with consensus reached on the importance of 18 statements. In the second round, 25 statements were presented: 22 feasibility statements, two revised statements that did not reach consensus on importance in the first round and referred to lack of data on AMR frequency and burden of CR *P. aeruginosa, A. baumannii*, *E. coli* and *K. pneumoniae* infections from Europe, and one new statement on lack of data on burden of drug-susceptible infections based on experts’ suggestions. In the second round, experts reached consensus on deprioritising research on the burden of infections by CR bacteria in Europe. This decision was based on the observation that while CR bacteria have a high prevalence in southern Europe, the overall prevalence across Europe remains relatively low, limiting the feasibility of quantitative research. Knowledge gaps that were deemed feasible to address with future research are summarised in Box 1, stratified by PICO elements. Results of ranking statements by importance are provided in Supplementary Table S8.

Box 1 **Prioritised knowledge gaps on antimicrobial resistance burden to be addressed in future antimicrobial resistance research, informed by systematic reviews and rounds of Delphi questionnaires, organised by PICO (patients, intervention, comparison, outcome) elements**

**Patient population**

• Prevalence, incidence density (per patient-days or population) and resistance proportions, per pathogen, for the following drug-resistant infection types: bloodstream, urinary tract, and surgical site infections.

• Prevalence, incidence density (per patient-days or population) and resistance proportions of drug-resistant infections per pathogen for the following risk groups: transplant patients, surgical patients, elderly people, and patients with haemato-oncological malignancies.

• Health and economic burden of drug-resistant infections, per pathogen, for the following patient risk groups: surgical patients, patients with haemato-oncological malignancies, and paediatric populations.

**Comparator**

In studies assessing the impact of drug-resistant infections on health and economic outcomes, a comparison of patients with drug-resistant infections to patients with no infection is needed, in addition to the more frequently reported comparison to patients with drug-susceptible infections.

**Study outcomes**

• Better quality estimates of excess mortality and length of hospital stay in patients with bloodstream infections for 3GCR *Escherichia coli,* MRSA and VRE.^a^

• Health outcomes by type of drug-resistant infection (in order of priority): (i) bloodstream infection (mortality, duration of hospitalisation in ICU following an infection and clinical failure/recurrence), (ii) urinary tract infection (mortality and clinical failure/recurrence), (iii) pneumonia (mortality, duration of hospitalisation in ICU following an infection and clinical failure/recurrence), and (iv) surgical site infection (mortality, duration of hospitalisation in ICU following an infection and clinical failure/recurrence).

• Non-fatal health outcomes and (any) economic outcomes in patients with bloodstream infections for 3GCR *E. coli* and MRSA.^a^

• Economic burden of drug-resistant infections, per pathogen, measured by resource use associated with the drug-resistance, which should include in order of priority: (i) length of hospital stay (by ward/specialty), (ii) antibiotic treatment courses, (iii) utilisation of diagnostics, and (iv) need for non-pharmaceutical interventions.

3GCR: third generation cephalosporin-resistant; ICU: intensive care unit; MRSA: meticillin-resistant *S. aureus*; PICO: patients, intervention, comparison, outcome; VRE: vancomycin resistant *Enterococcus faecium.*

^a^ Evidence was available on these specific pathogen-infection combinations, but was of low quality.

## Target populations and relevant clinical and economic outcomes

Specific high-risk groups were identified as a priority for future studies, including surgical patients and patients with haemato-oncological malignancies for studies on both AMR frequency measures and health-economic outcomes (Box 1). To assess the health burden of AMR, a comparator group of patients with no infection was selected as a research priority in addition to those with susceptible infections. Experts commented that both comparisons are essential as they address different questions. For outcomes per infection type, all-cause mortality was prioritised for all infection types, given it is objective and relatively easy to assess. Non-fatal outcomes were considered as a second priority for less severe infections as well as for bloodstream infections (BSIs), specifically selecting length of intensive care unit (ICU) stay and clinical failure/infection recurrence. Regarding economic outcomes, experts agreed that the resource use (e.g. bed-days, medical devices use, health supplies, services and equipment) should be prioritised because of their larger external validity across healthcare systems and economies compared to crude costs. The experts underlined the importance of better data for the clinical and economic impact of drug-resistant BSIs, specifically for MRSA, VRE and 3GCR *E. coli*.

## Pathogen-infection ranking

Based on the pathogen-infection ranking statements, BSIs were identified as the first AMR research priority, focusing on 3GCR *K. pneumoniae*, CR *K. pneumoniae* and CR *A. baumannii* (Table). Surgical site infections (SSIs) were identified as the second priority, where MRSA was the leading research focus. Urinary tract infections (UTIs) were included as the third, feasible priority with a focus on 3GCR *E. coli*. For respiratory tract infections (RTIs), pathogen prioritisation was also determined. However, experts found that filling this knowledge gap is unfeasible due to diagnostic challenges, difficulties in distinguishing colonisation from infection and sample size constrains for this infection type (Table).

**Table:** Priority infection-pathogen combinations for antimicrobial resistance burden studies on frequency, health and economic outcomes informed by Delphi rounds

Infection type (ordered by importance)	Pathogens (ordered by importance)
Bloodstream infections	(i) Third generation cephalosporin-resistant *Klebsiella pneumoniae* (ii) Carbapenem-resistant *K. pneumoniae* (iii) Carbapenem-resistant *Acinetobacter baumannii*
Surgical site infections	(i) Meticillin-resistant *Staphylococcus aureus* (ii) Third generation cephalosporin-resistant *K. pneumoniae* (iii) Third generation cephalosporin-resistant *Escherichia coli*
Respiratory tract infections (limited feasibility)	(i) Carbapenem-resistant *Pseudomonas aeruginosa* (ii) Carbapenem-resistant *K. pneumoniae* (iii) Carbapenem-resistant *A. baumannii*
Urinary tract infections	(i) Third generation cephalosporin-resistant *E. coli* (ii) Carbapenem-resistant *E. coli* (iii) Third generation cephalosporin-resistant *K. pneumoniae*

## Challenging research agenda items unfeasible to achieve in the near future

Further research agenda items were considered important yet unfeasible to address, in the near future, for reasons related to infection type and setting (Box 2). For example, lack of testing for community onset UTI, the need for outpatient follow-up to detect SSI and diagnostic challenges for RTIs. Experts stated additional possible challenges concerning specific settings, for example comprehensive AMR surveillance in European countries with limited resources. They also considered factors related to specific patient populations, e.g. operational issues for research on paediatric patients, the need for informed consent from parents and difficulty obtaining a large enough sample size. Limited access to data was also highlighted as a challenge, especially for evaluating economic outcomes associated with AMR. Challenges to data collection and standardising definitions were noted, specifically for non-fatal outcomes such as clinical failure and physical debilitation/deconditioning.

Box 2 **Research priority items that experts deemed as unfeasible to achieve in the near future informed by two Delphi rounds**

**Patient population**

• High quality frequency measures (prevalence, incidence density and resistance proportions), clinical and economic burden of drug-resistant infections, per pathogen, from European countries with limited resources.

• Prevalence, incidence density (per patient-days or population) and resistance proportions of drug-resistant infections, per pathogen, in neonates and children.

• Prevalence, incidence density (per patient-days or population) and resistance proportions, per pathogen, for patients with drug-resistant respiratory tract infection.

• Health and economic burden of drug-resistant infection per pathogen for elderly patients, neonates and immunocompromised patients.

• Better quality studies on excess mortality and length of stay for patients with MRSA pneumonia.^a^

**Study outcomes**

• Clinical failure/ recurrence of infection and physical debilitation/ deconditioning^b^, per pathogen, for drug-resistant pneumonia.

• Physical debilitation/deconditioning, per pathogen, for drug-resistant urinary tract infections.

• Economic burden measured as work absenteeism due to a drug-resistant infection, per pathogen.

^a^ Evidence was available on these specific pathogen-infection combinations, but was of low quality.
^b^ Physical debilitation or deconditioning refers to the decline in physical strength, endurance and overall functional ability resulting from illness.

## Improving data quality and harmonisation

Recommendations to improve data quality and harmonisation of future studies based on the Delphi consensus are summarised in Box 3. One of the key challenges in data aggregation was the use of different mortality endpoints in studies on clinical outcomes [11]. Experts prioritised 30-day all-cause mortality with post-discharge follow-up as the endpoint since it is most relevant to patients. However, its feasibility might be limited depending on the availability of post-discharge follow-up. Additional elements based on structured discussion within the steering committee are also provided in Box 3, such as general knowledge gaps, harmonised definitions for infection onset and prioritising the use of date of culture as onset date as the most feasible and relevant measures.

Box 3 **Recommendations to improve quality of future studies on the burden of antimicrobial resistance**

**A. Recommendations informed by two Delphi rounds**

***Mortality associated with drug-resistant infections***

Timepoints for mortality assessment after infection onset should be standardised.

Recommended timepoints, ordered by priority are:

- 30-day mortality with post-discharge follow-up;
- 14-day mortality with post-discharge follow-up;
- 30-day mortality without post-discharge follow-up;
- In-hospital mortality without time limit;
- 14-day mortality without post-discharge follow-up.

***Economic outcomes associated with drug-resistant infections***

Characteristics of the included patient population should be reported, e.g. frequency of comorbidities, to better understand representativeness and external validity of the study findings.

***Comparator group***

In AMR burden studies, compare clinical and/or economic outcomes between patients with drug-resistant infections to two comparator groups (patients with drug-susceptible infections and patients with no infection). It is important to add a comparison of outcomes of patients with drug-susceptible infections to patients with no infection to estimate the burden of susceptible infections.

***Granular data***

When AMR burden data (frequency measures, clinical and economic outcomes) are reported for drug-resistant Enterobacterales infections, and the sample size of the study is large enough, data disaggregated at pathogen level should be reported (i.e. separate burden estimates for *Escherichia coli*, *Klebsiella pneumoniae* and other pathogens).

***Denominator data***

Surveillance studies reporting drug resistance percentages should always report an estimation of the underlying population size to enable estimates of prevalence and incidence.

**B. Recommendations informed by structured discussions by the steering committee**

***General knowledge gaps***

- Studies are encouraged to determine the AMR frequency and burden in community acquired infections.
- Studies are encouraged to determine the AMR frequency and burden in healthcare-acquired infections in long-term care facilities.

***Measurements and reporting***

- Incidence densities (per patient-days or population) should be considered the more important frequency measure than prevalence/resistance proportions.
- In studies assessing burden of AMR, transparent reporting of the timing and assessment method of clinical failure and/or recurrence is needed. A time window of 30 days after infection should be applied for bloodstream infections, urinary tract infections and pneumonia.
- When reporting on ICU admission in a study on AMR burden, it is critical to define whether it was measured as a confounder and/or as a consequence of the infectious episode.
- In studies evaluating AMR burden in hospital-acquired infections, the time between admission and infection onset should be taken into account in the study design and/or data analysis, to avoid immortal-time bias.
- In observational studies evaluating AMR burden, infection onset should be clearly defined and reported. The recommended timepoints, ordered by priority, are time of culture collection for microbiological assessment, time of symptom onset, and time of initiation of empirical antibiotic therapy.
- In studies evaluating AMR burden on length of hospital stay for hospital-acquired infections, length of stay should only be measured following infection onset.

***Bias and confounding***

- Studies evaluating AMR burden confounding should generate meaningful burden estimates, minimally including comorbidities and a measure of severity of disease before infection onset.
- For surveillance studies reporting drug resistance percentages, prevalence or incidence estimates, any potential selection bias(es), such as frequent culturing after treatment failure or overrepresentation of specific patient groups, should be reported.

AMR: antimicrobial resistance; ICU: intensive care unit; PrIMAVeRa: Predicting the Impact of Monoclonal Antibodies & Vaccines on Antimicrobial Resistance.

## Research agenda for AMR burden research priorities in context

We present an agenda for prioritising future AMR burden research in humans to guide target and population selection for new pathogen-specific preventive and treatment strategies, specifically vaccines and mAbs, to efficiently contain or reduce the burden of AMR in Europe. Our exercise characterised important knowledge gaps and biases in current AMR literature from Europe and provides prioritisation of future research on infections and specific pathogens for each infection type. The provided agenda considers current data availability and the importance and feasibility of future research in Europe. It is important to note, however, that regional variations in drug resistance levels and availability of clinical evidence on AMR burden can result in different national priorities compared to European-level priorities. Also, while the largest amount of evidence was found for BSIs due to VRE, MRSA and 3GCR *E. coli*, experts agreed that better quality evidence is still needed. In addition, paucity of any data on community-acquired infections, and those acquired in long-term health-care facilities were recognised as important, general knowledge gaps by the PrIMAVeRa experts.

Antimicrobial resistance burden data stratified by pathogen, infection and patient risk-group is not only key for pathogen-specific interventions such as vaccines or mAbs, but has wider value. In the latest global burden of disease study [22], lack of detailed data limited reliable estimates of the AMR burden due to mortality risks being kept equal for age, sex and infection type [23]. In the 2023 ECDC AMR report, lack of specific burden estimates was also noted [24]. As such, implementation of the current research agenda can provide a stronger evidence base for future European AMR burden estimates by providing harmonised data on mortality risks per pathogen-infection combination and for relevant high-risk patient groups, resulting in more actionable results.

The selection of pathogens included in this Delphi exercise and in its supportive systematic reviews were based on multiple criteria, including being listed on the 2017 WHO PPL. This WHO list was developed to prioritise R&D of new antibiotics from a global perspective and was based on criteria including associated burden and lack of available treatments [15]. The WHO list was recently updated [25], providing separate ‘critical’ priority statuses for CR and 3GCR Enterobacterales, and downgrading CR *P. aeruginosa* as ‘high’ rather than ‘critical’ priority, while MRSA remained ‘high’ priority. Some new pathogens were added mostly due to their burden in low- and middle-income countries. As such, our selection of pathogens would not have changed based on the new BPPL. The WHO report further underlines the importance of AMR surveillance and the significant gaps in data on pathogen trends and disease burden, advocating for investments in improved data collection and analysis.

The WHO has also recently published a global research agenda for AMR in human health to inform policy by 2030 [26]. This agenda highlights various priority areas to reduce the burden of AMR, which includes assessment of the potential impact of vaccination for prevention. It reiterates the need for epidemiological studies assessing prevalence, mortality, morbidity and impact of infections caused by resistant pathogens, with emphasis on data disaggregated by subpopulations. Our findings confirm that in Europe, little reliable data on AMR burden exists for the ‘critical’ and ‘high’ priority pathogens and we provide a detailed list of research priorities that could improve the evidence base for future priority lists. In addition, our current work emphasises the importance of a more granular priority list, indicating the importance of drug-resistant pathogens per infection type and at-risk populations to better inform potential preventive and treatment strategies. This would be essential to ensure that resources are allocated to areas with the highest potential impact on AMR.

Our exercise has notable strengths; the Delphi process, which is considered the preferred consensus-based method for outcome selection, was informed by comprehensive, parallel systematic reviews of the literature, screening 19,500 publications and data sources and relying on 450 published studies and surveillance networks [10-12]. However, there are also some limitations to consider. First, the Delphi response rate was relatively low at 62%, and included 24 AMR experts, with a lack of experts from central or eastern Europe. Nevertheless, we achieved the predefined number of responses, with a diverse expert panel, with regards to gender, age and expertise. Second, ranking the problem statements in the Delphi questionnaire was challenging for the experts. Detailed pairwise rankings of multiple items (PAPRIKA method) could have provided more precise ranking results. However, with the large number of items that needed to be considered, this would have made the Delphi exercise very time-consuming and would have possibly introduced bias through reduced response rates. Third, interviews or focus group discussions were not performed, limiting experts’ input in the Delphi process. However, open text fields frequently used by the experts allowed for additional input, which was integrated in subsequent rounds. Then, the value of information techniques using health-economic models could have augmented this exercise by taking limited research funding budgets into account. However, this would require experts to be able to assess the added value of perfect knowledge in each specific setting. Since our focus was not on health technology assessment experts, and we had a long list of items to prioritise, we felt the Delphi methodology was a more effective method to obtain a prioritisation ranking. Finally, this exercise was specifically focused on research that could inform strategies aimed at humans, like vaccines and mAbs, to reduce the burden of AMR. Evidence and research priorities for other strategies under the One Health umbrella have not been assessed.

## Conclusion

We developed an AMR research agenda based on knowledge gaps identified through prior systematic reviews, and a Delphi exercise. The agenda provides guidance for future research to support data-driven selection of target pathogens and populations for new preventive and treatment strategies, specifically vaccines and mAbs, to effectively address the human AMR burden in Europe. These research priorities are also relevant to improve the evidence base for future AMR burden estimates. The listed research priorities can help guide funders and researchers in the field to focus their research and fill the most important AMR knowledge gaps.

## Supplementary Data

333000 Supplementary Material 1

## Supplementary Data

367000 Supplementary Material 2

## References

[1] Jansen KU Anderson AS . The role of vaccines in fighting antimicrobial resistance (AMR). Hum Vaccin Immunother. 2018;14(9):2142-9. 10.1080/21645515.2018.1476814 29787323 PMC6183139

[2] Zurawski DV McLendon MK . Monoclonal Antibodies as an Antibacterial Approach Against Bacterial Pathogens. Antibiotics (Basel). 2020;9(4):155. 10.3390/antibiotics9040155 32244733 PMC7235762

[3] Klugman KP Black S . Impact of existing vaccines in reducing antibiotic resistance: Primary and secondary effects. Proc Natl Acad Sci USA. 2018;115(51):12896-901. 10.1073/pnas.1721095115 30559195 PMC6304973

[4] Andrejko K Ratnasiri B Hausdorff WP Laxminarayan R Lewnard JA . Antimicrobial resistance in paediatric Streptococcus pneumoniae isolates amid global implementation of pneumococcal conjugate vaccines: a systematic review and meta-regression analysis. Lancet Microbe. 2021;2(9):e450-60. 10.1016/S2666-5247(21)00064-1 34485957 PMC8410609

[5] Wilcox MH Gerding DN Poxton IR Kelly C Nathan R Birch T Bezlotoxumab for Prevention of Recurrent Clostridium difficile Infection. N Engl J Med. 2017;376(4):305-17. 10.1056/NEJMoa1602615 28121498

[6] Mohamed MFH Ward C Beran A Abdallah MA Asemota J Kelly CR . Efficacy, Safety, and Cost-effectiveness of Bezlotoxumab in Preventing Recurrent Clostridioides difficile Infection: Systematic Review and Meta-analysis. J Clin Gastroenterol. 2024;58(4):389-401. 10.1097/MCG.0000000000001875 37395627

[7] Frost I Sati H Garcia-Vello P Hasso-Agopsowicz M Lienhardt C Gigante V The role of bacterial vaccines in the fight against antimicrobial resistance: an analysis of the preclinical and clinical development pipeline. Lancet Microbe. 2023;4(2):e113-25. 10.1016/S2666-5247(22)00303-2 36528040 PMC9892012

[8] Troisi M Marini E Abbiento V Stazzoni S Andreano E Rappuoli R . A new dawn for monoclonal antibodies against antimicrobial resistant bacteria. Front Microbiol. 2022;13:1080059. 10.3389/fmicb.2022.1080059 36590399 PMC9795047

[9] Oyston P Robinson K . The current challenges for vaccine development. J Med Microbiol. 2012;61(Pt 7):889-94. 10.1099/jmm.0.039180-0 22322337

[10] Pezzani MD Arieti F Rajendran NB Barana B Cappelli E De Rui ME Frequency of bloodstream infections caused by six key antibiotic-resistant pathogens for prioritization of research and discovery of new therapies in Europe: a systematic review. Clin Microbiol Infect. 2024;30(Suppl 1):S4-13. 10.1016/j.cmi.2023.10.019 38007387

[11] Hassoun-Kheir N Guedes M Ngo Nsoga MT Argante L Arieti F Gladstone BP A systematic review on the excess health risk of antibiotic-resistant bloodstream infections for six key pathogens in Europe. Clin Microbiol Infect. 2024;30(Suppl 1):S14-25. 10.1016/j.cmi.2023.09.001 37802750

[12] Kingston R Vella V Pouwels KB Schmidt JE Abdelatif El-Abasiri RA Reyna-Villasmil E Excess resource use and cost of drug-resistant infections for six key pathogens in Europe: a systematic review and Bayesian meta-analysis. Clin Microbiol Infect. 2024;30(Suppl 1):S26-36. 10.1016/j.cmi.2023.12.013 38128781

[13] The Epidemiology Network (EPI-NET). PrIMAVeRa: Predicting the impact of monoclonal antibodies and vaccines on the burden of antimicrobial resistance 2022-2026. 2023. Available from: https://epi-net.eu/primavera/about/

[14] Centers for Disease Control and Prevention (CDC). Antibiotic Resistance Threats in the United States, 2019. Atlanta: CDC; 2019.Available from: https://www.cdc.gov/antimicrobial-resistance/data-research/threats/index.html

[15] Tacconelli E Carrara E Savoldi A Harbarth S Mendelson M Monnet DL Discovery, research, and development of new antibiotics: the WHO priority list of antibiotic-resistant bacteria and tuberculosis. Lancet Infect Dis. 2018;18(3):318-27. 10.1016/S1473-3099(17)30753-3 29276051

[16] Cassini A Högberg LD Plachouras D Quattrocchi A Hoxha A Simonsen GS Attributable deaths and disability-adjusted life-years caused by infections with antibiotic-resistant bacteria in the EU and the European Economic Area in 2015: a population-level modelling analysis. Lancet Infect Dis. 2019;19(1):56-66. 10.1016/S1473-3099(18)30605-4 30409683 PMC6300481

[17] European Centre for Disease Prevention and Control (ECDC). Antimicrobial resistance in the EU/EEA (EARS-Net) - Annual Epidemiological Report. Stockholm: ECDC; 2019. Available from: https://www.ecdc.europa.eu/en/publications-data/surveillance-antimicrobial-resistance-europe-2019

[18] Robinson KA, Akinyede O, Dutta T, Ivey Sawin V, Tianjing L, Spencer MR, et al. Framework for Determining Research Gaps During Systematic Review: Evaluation. Rockville (MD): Agency for Healthcare Research and Quality (US); 2013. Report No.: 13-EHC019-EF. PMID:23487868 23487868

[19] Niederberger M Spranger J . Delphi Technique in Health Sciences: A Map. Front Public Health. 2020;8:457. 10.3389/fpubh.2020.00457 33072683 PMC7536299

[20] Wang LM Cravo Oliveira Hashiguchi T Cecchini M . Impact of vaccination on carriage of and infection by antibiotic-resistant bacteria: a systematic review and meta-analysis. Clin Exp Vaccine Res. 2021;10(2):81-92. 10.7774/cevr.2021.10.2.81 34222121 PMC8217572

[21] Stewardson AJ Allignol A Beyersmann J Graves N Schumacher M Meyer R The health and economic burden of bloodstream infections caused by antimicrobial-susceptible and non-susceptible Enterobacteriaceae and Staphylococcus aureus in European hospitals, 2010 and 2011: a multicentre retrospective cohort study. Euro Surveill. 2016;21(33):30319. 10.2807/1560-7917.ES.2016.21.33.30319 27562950 PMC4998424

[22] Antimicrobial Resistance Collaborators . Global burden of bacterial antimicrobial resistance in 2019: a systematic analysis. Lancet. 2022;399(10325):629-55. 10.1016/S0140-6736(21)02724-0 35065702 PMC8841637

[23] de Kraker MEA Harbarth S . Global burden of antimicrobial resistance: essential pieces of a global puzzle. Lancet. 2022;399(10344):2347. 10.1016/S0140-6736(22)00940-0 35753335

[24] European Centre for Disease Prevention and Control (ECDC). Antimicrobial resistance surveillance in Europe 2023 - 2021 data. Stockholm: ECDC; 2023. Available from: https://www.ecdc.europa.eu/en/publications-data/antimicrobial-resistance-surveillance-europe-2023-2021-data

[25] World Health Organization (WHO). WHO Bacterial Priority Pathogens List 2024, bacterial pathogens of public health importance to guide research, development and strategies to prevent and control antimicrobial resistance. Geneva: WHO; 2024. Available from: https://www.who.int/publications/i/item/9789240093461

[26] World Health Organization (WHO). Global research agenda for antimicrobial resistance in human health. Geneva: WHO; 2023. Available from: https://www.who.int/publications/m/item/global-research-agenda-for-antimicrobial-resistance-in-human-health

