# Supplementary material for: Expert consensus on antimicrobial resistance research priorities to focus development and implementation of antibacterial vaccines and monoclonal antibodies
Source: Euro Surveill. 2024 Nov 21;29(47):2400212. doi: 10.2807/1560-7917.ES.2024.29.47.2400212 (PMC11583311; doi:10.2807/1560-7917.ES.2024.29.47.2400212)

Supplementary Material 1

This supplementary material is hosted by *Eurosurveillance* as supporting information alongside the article 'Expert consensus on antimicrobial resistance research priorities to focus development and implementation of antibacterial vaccines and monoclonal antibodies', on behalf of the authors, who remain responsible for the accuracy and appropriateness of the content. The same standards for ethics, copyright, attributions and permissions as for the article apply. Supplements are not edited by *Eurosurveillance* and the journal is not responsible for the maintenance of any links or email addresses provided therein.

Table of contents

Table S1 - Resistant pathogen-infection combinations evaluated in the systematic reviews.......... 2

Table S2 - Knowledge gaps mapping per PICO element identified in the systematic reviews 2

Table S3 - Number of studies identified in the systematic review reporting frequency measures (resistance rate, prevalence, and incidence density) for selected pathogen-infection combinations 3

Table S4 - Number of studies identified in the systematic review reporting clinical outcomes for selected pathogen-infection combinations.......... 3

Table S5 –Number of studies identified in the systematic review reporting economic outcomes for selected pathogen-infection combinations.......... 4

Consensus approach in Delphi survey.......... 5

Table S6 – Demographics and qualifications of experts who participated in each Delphi consensus round.......... 6

Table S7 – Problem statements that achieved consensus at each Delphi stage.......... 7

Table S8 – Results of the ranking statements in the Delphi study (N=24 experts).......... 8

Figure S1 - Study flow diagram 10

Table S1 - Resistant pathogen-infection combinations evaluated in the systematic reviews

Pathogen	Relevant resistance profile	Infection types
Acinetobacter baumannii	Carbapenems	BSI, RTI, UTI
Pseudomonas aeruginosa	Carbapenems	BSI, RTI, UTI, SSI
Escherichia coli	Carbapenems / 3rd generation cephalosporins	BSI, RTI, UTI, SSI, IAI*
*Klebsiella pneumoniae**	Carbapenems / 3rd generation cephalosporins	BSI, RTI, UTI, SSI, IAI*
Staphylococcus aureus	Methicillin	BSI, RTI, SSTI, SSI
Enterococcus faecium	Vancomycin	BSI, UTI, IAI*

BSI - bloodstream infections, RTI - respiratory tract infections, UTI - urinary tract infections, SSTI - skin and soft tissue infections, SSI - surgical site infections, IAI - intraabdominal infections, *IAI were evaluated as polymicrobial infection

Table S2 - Knowledge gaps mapping per PICO element identified in the systematic reviews

Element	Interpretation
Patients	Evaluation whether specific infection types were reported per pathogen, and whether results were available stratified by age groups (adults/children), high-risk populations (as defined in systematic reviews) and setting (hospital/community/long term care facility)
Exposures	Predefined drug-resistance patterns considered (Table S1)
Comparator (relevant only for economic and health outcomes)	Comparators included patients with susceptible infection and with no infection
Outcomes	1. Frequency measures (prevalence, incidence and resistance proportion)<br>2. Health risks (all-cause and infection related mortality, recurrence of infection, clinical failure, organ failure and others)<br>3. Economic resource utilization (length of stay, re-admission, resource use and specific costs)

Table S3 - Number of studies identified in the systematic review reporting frequency measures (resistance rate, prevalence, and incidence density) for selected pathogen-infection combinations

**Resistant pathogen/infection**	**BSI**	**RTI/ VAP**	**UTI**	**SSTI**	**SSI**	**IAI***
Carbapenem resistant *Pseudomonas aeruginosa*	8	3/5	8		0	
Carbapenem resistant *Acinetobacter baumannii*	5	4/3	1			
3rd gen cephalosporin resistant *Escherichia coli*	17	9/4	34		2	6
Carbapenem resistant *Escherichia coli*	10	4/1	11		0	2
3rd gen cephalosporin resistant *Klebsiella pneumoniae*	115	5/5	13		1	4
Carbapenem resistant *Klebsiella pneumoniae*	14	3/3	4		2	2
Methicillin resistant *Staphylococcus aureus* (MRSA)	31	18/5		12	8	
Vancomycin resistant *Enterococcus faecium* (VRE)	10		4			2

BSI - bloodstream infections, RTI - respiratory tract infections, UTI - urinary tract infections, SSTI - skin and soft tissue infections, SSI - surgical site infections, IAI - intraabdominal infections. *For IAI – polymicrobial infections were also considered due to the nature of this infection type

Table S4 - Number of studies identified in the systematic review reporting clinical outcomes for selected pathogen-infection combinations

Resistant pathogen/infection	BSI	RTI	UTI	SSTI	SSI	IAI*
Carbapenem resistant *Pseudomonas aeruginosa*	7	2	1		0	
Carbapenem resistant *Acinetobacter baumannii*	7	2	1		0	
3rd gen cephalosporin resistant *Escherichia coli*	13	1	6		0	0
Carbapenem resistant *Escherichia coli*	0	0	0		0	0
3rd gen cephalosporin resistant *Klebsiella pneumoniae*	3	0	3		0	0
Carbapenem resistant *Klebsiella pneumoniae*	5	0	0		0	0
3rd gen cephalosporin resistant *Enterobacterales*	2	0	2		0	0
Methicillin resistant *Staphylococcus aureus* (MRSA)	16	11		16	6	0
Vancomycin resistant *Enterococcus faecium* (VRE)	14		2			0

BSI - bloodstream infections, RTI - respiratory tract infections, UTI - urinary tract infections, SSTI - skin and soft tissue infections, SSI - surgical site infections, IAI - intraabdominal infections. *For IAI – polymicrobial infections were also considered due to the nature of this infection type

Table S5 –Number of studies identified in the systematic review reporting economic outcomes for selected pathogen-infection combinations

Resistant pathogen/infection	BSI	RTI	UTI	SSTI	SSI	IAI*	Un-specified
Carbapenem resistant *Pseudomonas aeruginosa*	0	0	0		0		1
Carbapenem resistant *Acinetobacter baumannii*	0	0	0				0
3rd gen cephalosporin resistant *Escherichia coli*	9	1	4		0	1	2
Carbapenem resistant *Escherichia coli*	1	0	0		0	0	0
3rd gen cephalosporin resistant *Klebsiella pneumoniae*	2	1	3		0	0	2
Carbapenem resistant *Klebsiella pneumoniae*	0	1	0		0	0	0
Methicillin resistant *Staphylococcus aureus* (MRSA)	9	5		2	2		5
Vancomycin resistant *Enterococcus faecium* (VRE)	2		0			0	1

BSI - bloodstream infections, RTI - respiratory tract infections, UTI - urinary tract infections, SSTI - skin and soft tissue infections, SSI - surgical site infections, IAI - intraabdominal infections. *For IAI – polymicrobial infections were also considered due to the nature of this infection type

Consensus approach in Delphi survey

- Experts were asked to score their agreement with the provided statements based on a Likert scale (1=strongly disagree, 2=moderately disagree, 3=neither agree nor disagree, 4=moderately agree, 5= strongly agree, and an additional option: No expertise in this field).
- Consensus definitions: In statements seeking agreement on a Likert scale, median scores, and interquartile ranges (IQRs), and the percentage of experts scoring ≥ 4, were calculated in each round to indicate levels of consensus and agreement, respectively. Thresholds and definitions of consensus were based on values used in previous studies:
    - "Consensus" median score ≥ 4 and percentage of agreement ≥80%
    - "Low agreement" median score< 3 or percentage of agreement < 60%
    - "Intermediate agreement" all the other statements.
- Statements with low agreement were dropped and those with intermediate agreement were revised for a subsequent round.
- For priority statements by importance, those with the highest importance (top half) were carried on to subsequent rounds. In the case of ties, multiple statements were considered equally important.
- For feasibility assessment, the statement in the lowest quartile of ranking were deemed unfeasible. Statements scoring equal to the feasibility threshold were considered feasible.

Table S6 – Demographics and qualifications of experts who participated in each Delphi consensus round

	**Round one** **N=24** (%)	**Round two** **N=19** (%)
Gender		
Male	14 (58.3)	11 (57.9)
Female	8 (33.3)	7 (36.8)
Not reported	2 (8.3)	1 (5.3)
Age group		
<30 years	0 (0.0)	0 (0.0)
30-40 years	7 (29.2)	6 (31.6)
41-50 years	5 (20.8)	4 (21.1)
51-60 years	8 (33.3)	7 (36.8)
>60 years	2 (8.3)	2 (10.5)
Not reported	2 (8.3)	0 (0.0)
Years of expertise in AMR		
<5 years	1 (4.2)	0 (0.0)
6-10 years	7 (29.2)	6 (31.6)
11-20 years	7 (29.2)	7 (36.8)
>20 years	6 (25.0)	5 (26.3)
Not reported	3 (12.5)	1 (5.3)
Employer in Europe		
Yes	16 (66.7)	15 (78.9)
No	5 (20.8)	4 (21.1)
Not reported	3 (12.5)	0(0.0)
Area(s) of expertise*		
Infectious diseases epidemiology	17 (70.8)	16 (84.2)
Health economics and/or health financing	4 (16.7)	3 (15.8)
Healthcare provider	3 (12.5)	3 (15.8)
Public health/Health policy/Global health	7 (29.2)	6 (31.6)
Clinical AMR research funding	1 (4.2)	1 (5.3)
Type(s) of organization*		
Academic institution	14 (58.3)	13 (68.4)
Funding agency	0 (0.0)	0 (0.0)
Healthcare provider facility	5 (20.8)	5 (26.3)
Non-governmental organization	3 (12.5)	1 (5.3)
Governmental/public health organization	2 (8.3)	2 (10.5)
Pharma industry	4 (16.7)	3 (15.8)

* Each expert could state multiple expertise and/or organization types

Table S7 – Problem statements that achieved consensus at each Delphi stage

No.	Statement	Agreement on importance	Agreement on Feasibility	Consensus on importance
1	There is lack of data regarding the burden of AMR (clinical and economic impact) within pediatric populations	94.1%	77.8%	Consensus in 1st round
2	There is lack of data on the burden of AMR (frequency, clinical and economic impact) from Eastern and Central European countries	100.0%	53.0%	Consensus in 1st round
3	There is lack of data on health and/or economic burden of AMR for carbapenem resistant infections, caused by *P. aeruginosa*, *A. baumannii*, *E. coli* and *K. pneumoniae* from Europe	77.8%	72.2%	Revised for 2nd round and dropped
4	Most AMR burden studies compare clinical and/or economic outcomes between patients with drug-resistant infections and patients with drug-susceptible infections (attributable burden), indicating the preventable burden of drug resistance. AMR burden studies comparing clinical and economic outcomes between patients with drug-resistant infections and patients without an infection (associated burden) are very rare, while this indicates the preventable burden of completely eliminating drug-resistant infections	90.0%	83.3%	Consensus in 1st round
8	Very few studies report on the economic outcomes associated with AMR, and those that do, tend to report crude costs (*e.g.* cost in dollars) rather than resource use (e.g. number of CT scans, or number of courses of antibiotics). Clinical studies on the burden of AMR should include estimates of resource use associated with drug-resistance for a minimal set of items that can then be linked to unit costs.	85.0%	76.5%	Consensus in 1st round
16	When frequency measures (incidence/ prevalence) of resistant Enterobacterales infections are reported and when the sample size of the study is large enough, it should include disaggregated data stratified by pathogen	88.2%	NA	Consensus in 1st round
17	When AMR burden data (clinical and economic outcomes) is reported for resistant Enterobacterales infections and when the sample size of the study is large enough, it should include disaggregated data stratified by pathogen	84.2%	NA	Consensus in 1st round
19	Surveillance studies reporting drug resistance percentages should always report an estimation of the size of the population from which the study sample was taken, to allow for prevalence and incidence estimates generation.	88.2%	50.0%	Consensus in 1st round
20	Studies assessing economic outcomes associated with AMR should report information on the characteristics of the included patient population, like frequency of comorbidities, to better understand representativeness and external validity.	94.7%	72.2%	Consensus in 1st round
21	In AMR burden studies comparing clinical and/or economic outcomes between patients with drug-resistant infections to two comparator groups (patients with drug-susceptible infections and patients without an infection), it is also important to compare the outcomes of patients with drug-susceptible infections to patients without an infection to estimate the burden of susceptible infections.	94.4%	94.4%	Added in 2nd round
22	There is lack of data on frequency measures (i.e., prevalence, incidence) of carbapenem resistant infections, caused by *P. aeruginosa*, *A. baumannii*, *E. coli* and *K. pneumoniae* in Europe	77.8%	89.5%	Revised in 2nd round and dropped

Table S8 – Results of the ranking statements in the Delphi study (N=24 experts)

No	Statement	Importance ranking*
5	Most important clinical outcomes for AMR burden assessment per infection type	BSI: mortality (n=20, 83.3%), days in ICU following infection (n=12, 50.0%), clinical failure/ recurrence/relapse (n=9, 37.5%)<br>UTI: clinical failure/ recurrence/relapse (n=14, 58.3%), mortality (n=10, 41.7%), physical debilitation/deconditioning (temporary and/or permanent) (n=11, 45.8%)<br>RTI: mortality (n=18, 75.0%), days in ICU following infection (n=14, 58.3%), clinical failure/ recurrence/relapse (n=8, 33.3%), physical debilitation/deconditioning (temporary and/or permanent) (n=8, 33.3%)<br>SSI: mortality (n=16, 66.7%), days in ICU following infection (n=14, 58.3%), clinical failure/ recurrence/relapse (n=11, 45.8%)<br>SSTI: mortality (n=10, 41.7%), physical debilitation/deconditioning (temporary and/or permanent) (n=13, 54.2%), clinical failure/ recurrence/relapse (n=12, 50.0%)<br>IAI: mortality (n=18, 78.3%), days in ICU following infection (n=15, 65.2%), acute organ failure (n=8, 34.8%)
6	Most important patients' risk groups for AMR frequency measures reporting	Elderly (some data n=14, 58.3%)<br>Neonates (some data n=11, 45.8%)<br>Surgical patients (some data n=11, 45.8%)<br>Children (some data n=10, 41.7%)<br>Transplanted patients (some data n=9, 37.5%)<br>Patients with hemato-oncological malignancies (some data n=8, 33.3%)
7	Most important patients' risk groups for AMR burden (economic and health outcomes) reporting	Elderly (n=16, 66.7%)<br>Neonates (n=13, 54.2%)<br>Surgical patients (n=12, 50.0%)<br>Immunocompromised patients (n=11, 45.8%)<br>Patients with hemato-oncological malignancies (n=9, 37.5%)
9	Most important economic outcome for AMR burden assessment (all infection types)	Treatments (n=15, 65.2%)<br>Length of stay (by ward or specialty) (n=14, 60.9%)<br>Interventions (n=14, 60.9 %)<br>Absence from work (n=12, 52.5%)<br>Diagnostics (n=10, 43.5 %)
10	Most important infection types for frequency measures reporting	By descending order: BSI, SSI, RTI, UTI, IAI, SSTI
11	Most important infection types for AMR burden (economic and health outcomes) reporting	By descending order: BSI, UTI, RTI, SSI, IAI, SSTI
12	Future research priorities for AMR burden in MRSA BSI	Need for future higher quality studies (low risk of bias) measuring the mortality and length of hospital stay of patients with MRSA BSI (n=8, 42.1%)<br>Need for future studies on health outcomes other than mortality and economic outcomes in terms of resource use associated with MRSA BSI (such as recurrence, organ failure, ICU admission, treatment, healthcare utilization) (n=6, 31.6%)
13	Future research on burden in VRE BSI	Need for higher quality future studies measuring mortality and excess length of stay of vancomycin resistant Enterococcus bloodstream infections in Europe (n=10, 52·6)

14	Future research on 3rd generation cephalosporin resistant *E. coli* BSI	Need for higher quality studies on mortality and excess length of stay of 3rd generation cephalosporin resistant E. coli bloodstream infections (n=9,47·4%)<br>Need for future studies on health outcomes other than mortality and resource use associated with 3rd generation cephalosporin resistant E. coli bloodstream infections, like recurrence, organ failure, ICU admission, treatment and healthcare utilization (n=7, 36·8%)
15	Future research on MRSA respiratory tract infections	Need for high quality studies on mortality and excess length of stay of methicillin-resistant S. aureus respiratory tract infections in Europe (n=11, 57·9%)
18	Most important mortality assessment time points	By descending order: 30-day mortality (after infection onset) with post discharge follow-up, 30-day mortality (after infection onset) without post discharge follow-up, 14-day mortality (after infection onset) with post discharge follow-up, 14-day mortality (after infection onset) without post discharge follow-up

*Percentage denotes number of experts selecting this item (n) divided by valid responses per statement, experts were asked to select 1-5 most important items (See supplement 2).

Figure S1 - Study flow diagram

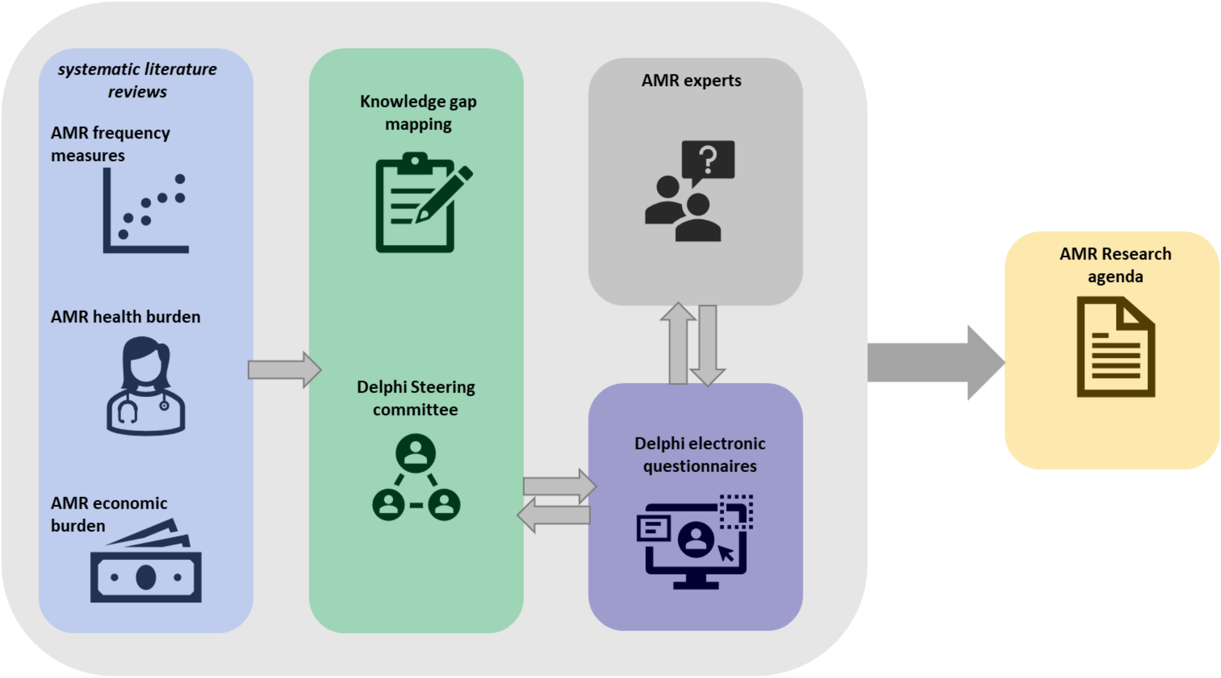

Supplement: Supplementary Material 1 [file 24-00212_DE_KRAKER_Supplementary_material_1.pdf]
