# Supplementary material for: Expert consensus on antimicrobial resistance research priorities to focus development and implementation of antibacterial vaccines and monoclonal antibodies
Source: Euro Surveill. 2024 Nov 21;29(47):2400212. doi: 10.2807/1560-7917.ES.2024.29.47.2400212 (PMC11583311; doi:10.2807/1560-7917.ES.2024.29.47.2400212)
Supplement: Supplementary Material 2 [file 24-00212_DE_KRAKER_Supplementary_material_2.pdf]

Supplementary Material 2

This supplementary material is hosted by *Eurosurveillance* as supporting information alongside the article ‘Expert consensus on antimicrobial resistance research priorities to focus development and implementation of antibacterial vaccines and monoclonal antibodies’, on behalf of the authors, who remain responsible for the accuracy and appropriateness of the content. The same standards for ethics, copyright, attributions and permissions as for the article apply. Supplements are not edited by *Eurosurveillance* and the journal is not responsible for the maintenance of any links or email addresses provided therein.

Round 1 – First questionnaire in the Delphi study

Expert elicitation process: Round 1

First, we will provide an overview of knowledge gaps for human AMR burden data based on three systematic reviews, focused on prevalence/incidence data, and associated clinical and economic outcomes from 1990-2023 (CRD 42022312795, CRD 2022322586, CRD 42022331400). Only pathogen-specific and infection-specific data were considered, e.*g.* outcomes associated with methicillin resistant *S. aureus* bloodstream infections.

Second, we will provide 25 statements indicating a specific or general research gap in human AMR burden research, for which we ask you to indicate whether you agree with the gap based on a Likert scale (1=strongly disagree, 2=moderately disagree, 3=neither agree nor disagree, 4=moderately agree, and 5= strongly agree, and an additional option: No expertise in this field). For some statements, you will need to rank the importance of the gap for different pathogens and/or infections. Finally, there will be room for you to add comments with regards to gaps that were overlooked, or other important messages. Based on pre-defined thresholds, new survey rounds will be created with more limited lists of statements that need to be prioritized or fine-tuned.

Specific objectives of this expert elicitation:

1. Evaluate the importance of identified knowledge gaps with regards to AMR burden, including frequency measures (*e.g.* prevalence, incidence), clinical and economic outcomes.
    - Fine-tuning of wording of, and elements included in, identified knowledge gaps with regards to AMR burden will be required.
2. Determine the feasibility of filling knowledge gaps with future research (based on factors such as study design required, size of study, costs of study, setting of study)
3. Determine the most important elements that should be considered in future research on AMR burden, including outcome definitions, common data dictionaries etc.
4. Identify additional relevant elements for future AMR burden research not yet considered based on expert opinion

Herein, we invite you to participate in the first round. It includes <u>20</u> gap statements for your consideration.
An overview of the questionnaire content can be downloaded here (pdf)

<u>Survey</u>

The survey includes 4 parts:

1. General information on the respondent
2. Statements about general knowledge gaps for AMR burden
3. Statements about specific knowledge gaps for AMR burden
4. Statements addressing harmonization of study definitions and methodology for future AMR burden studies

For all statements, the importance should be indicated on a Likert scale (1=strongly disagree…5= strongly agree).

Please keep in mind that we refer to the **<u>European setting</u>**, and that all research gaps should be considered in light of **<u>future health technology assessment of potential vaccines and monoclonal antibodies, or other pathogen-specific prevention or intervention strategies, that could reduce the burden of antimicrobial resistance</u>** (AMR).

<u>Participant information:</u>

- Institutional email address (optional)
- Country of practice
- Type of organization (check all that apply)
    - Academic institution
    - Funding agency
    - Healthcare provider
    - Non-governmental organization
    - Pharma industry
    - Other, please specify
- What are your main areas of expertise (check all that apply)?
    - Infectious diseases epidemiology
    - Health economics and/or health financing
    - Healthcare provider
    - Public health/Health policy/Global health
    - Research funding
    - Other
- How long have you been working in the AMR field?
    - <5 years
    - 6-10 years
    - 11-20 years
    - >20 years
- What is your sex?
    - Female
    - Male

- Prefer not to disclose
- What is your age group?
    - <30 years
    - 30-40 years
    - 41-50 years
    - 51-60 years
    - >60 years
    - Prefer not to disclose

General gaps

You will find 20 statements indicating a specific or general research gaps in human AMR burden research based on the systematic reviews, for which we ask you to indicate whether you agree with the gap based on a Likert scale (1=strongly disagree, 2=moderately disagree, 3=neither agree nor disagree, 4=moderately agree, and 5= strongly agree, and an additional option: No expertise in this field).

1. There is lack of data regarding the burden of AMR (clinical and economic impact) within pediatric populations

This is an important knowledge gap for evidence-based health technology assessment of potential vaccines and monoclonal antibodies to reduce the burden of AMR

1=strongly disagree
2=moderately disagree
3=neither agree nor disagree
4=moderately agree
5= strongly agree
6= No expertise in this field

Comment box:

2. There is lack of data on the burden of AMR (frequency, clinical and economic impact) from Eastern and Central European countries (Balkan, Poland, *etc*).

This is an important knowledge gap for health technology assessment of potential vaccines and monoclonal antibodies to reduce the burden of AMR

1=strongly disagree
2=moderately disagree
3=neither agree nor disagree
4=moderately agree
5= strongly agree
6= No expertise in this field

Comment box:

3. There is lack of data on the burden of AMR burden (frequency measures, clinical and economic impact) of carbapenem resistant infections, caused by *P. aeruginosa, A. baumannii, E. coli and K. pneumoniae*

This is an important knowledge gap for health technology assessment of potential vaccines and monoclonal antibodies to reduce the burden of AMR

1=strongly disagree
2=moderately disagree
3=neither agree nor disagree
4=moderately agree
5= strongly agree
6= No expertise in this field

Comment box:

4. Most AMR burden studies compare clinical and/or economic outcomes between patients with <u>drug-resistant</u> infections and patients with <u>drug-susceptible</u> infections (attributable burden), indicating the preventable burden of drug resistance. AMR burden studies comparing clinical and economic outcomes between patients with <u>drug-resistant</u> infections and patients <u>without an infection</u> (associated burden) are very rare, while this indicates the preventable burden of eliminating drug-resistant infections.

This is an important knowledge gap for health technology assessment of potential vaccines and monoclonal antibodies to reduce the burden of AMR
1=strongly disagree
2=moderately disagree
3=neither agree nor disagree
4=moderately agree
5= strongly agree
6= No expertise in this field

Comment box:

5. For the clinical burden of AMR, most studies report mortality as an outcome; other outcomes are not frequently reported.

Considering health technology assessment of potential vaccines and monoclonal antibodies to reduce the burden of AMR, what are

the most important outcomes that should be evaluated in future AMR burden research?

Please choose the **3 most important** outcomes per infection type (both hospital and community onset), you can suggest additional outcome (within the 3 outcomes limit).

5.1. Bloodstream infection (choose the 3 most important)

Mortality
Acute organ failure
Clinical failure/ recurrence/relapse
Days in ICU following infection
Physical debilitation/deconditioning (temporary and/or permanent)
Cognitive debilitation (temporary and/or permanent)
Other, please specify

5.2. *Urinary tract infection* (choose the 3 most important)

Mortality
Acute organ failure
Clinical failure/ recurrence/relapse
Days in ICU following infection
Physical debilitation/deconditioning (temporary and/or permanent)
Cognitive debilitation (temporary and/or permanent)
Other, please specify

5.3. *Respiratory tract infection* (choose the 3 most important)

Mortality
Acute organ failure
Clinical failure/ recurrence/relapse
Days in ICU following infection
Physical debilitation/deconditioning (temporary and/or permanent)
Cognitive debilitation (temporary and/or permanent)
Other, please specify

5.4. *Surgical site infections* (choose the 3 most important)

Mortality
Acute organ failure
Clinical failure/ recurrence/relapse
Days in ICU following infection
Physical debilitation/deconditioning (temporary and/or permanent)
Cognitive debilitation (temporary and/or permanent)
Other, please specify

5.5. *Skin and soft tissue infections* (choose the 3 most important)

Mortality
Cute organ failure
Clinical failure/ recurrence/relapse
Days in ICU following infection
Physical debilitation/deconditioning (temporary and/or permanent)
Cognitive debilitation (temporary and/or permanent)
Other, please specify

5.6. *Intra-abdominal infections* (choose the 3 most important)

Mortality
Acute organ failure
Clinical failure/ recurrence/relapse
Days in ICU following infection
Physical debilitation/deconditioning (temporary and/or permanent)
Cognitive debilitation (temporary and/or permanent)
Other, please specify

Comment box:

6. Frequency of drug resistant infection (prevalence, incidence) for specific risk groups is often not reported. It would be important to report frequency measures (*i.e. prevalence, incidence*) of infection for risk groups:

Considering health technology assessment of potential vaccines and monoclonal antibodies to reduce the burden of AMR, what are the most important risk groups that should be addressed in future AMR burden research?

Please choose the **5 most important risk groups**, specific data availability is noted in the brackets, you can add additional risk groups (within the 5 risk groups limit)

- Neonates (some data)
- Children (some data)
- Elderly (some data)
- Women (some data)

- Men (minimal/no data)
- Patients with diabetes (minimal/no data)
- Obese patients (minimal/no data)
- Pregnant women (minimal/no data)
- Transplanted patients (some data)
- Surgical patients (some data)
- Patients with hemato-oncological malignancies (some data)
- Patients with solid tumors (minimal/no data)
- Patients with chronic obstructive pulmonary disease (minimal/no data)
- Other, please specify

Comment box:

7. AMR burden (health and economic outcomes) for specific risk groups is rarely reported, and would be important:

Considering health technology assessment of potential vaccines and monoclonal antibodies to reduce the burden of AMR, what are the most important risk groups that should be addressed in future AMR burden research?

Please choose the **5 most important risk groups.** you can add additional risk groups (within the 5 risk groups limit)

- Neonates
- Elderly
- Women
- Men
- Patients with diabetes miletus
- Obese patients
- Intravenous drug users
- Pregnant women
- Immunocompromised patients
- Surgical patients
- Patients with hemato-oncological malignancies
- Patients with solid tumors
- Patients with chronic obstructive pulmonary disease
- Other, please specify

Comment box:

8. Very few studies report on the economic outcomes associated with AMR, and those that do tend to report crude costs rather than resource use. Clinical studies on the burden of AMR should include estimates of resource use associated with infection for a minimal set of items that can then be linked to unit costs.

Considering future health technology assessment of potential vaccines and monoclonal antibodies to reduce the burden of AMR, do you agree with this statement?

1=strongly disagree
2=moderately disagree
3=neither agree nor disagree
4=moderately agree
5= strongly agree
6= No expertise in this field

Comment box:

9. Economic outcomes associated with drug-resistant infections discussed in literature are often limited to excess length of stay or total costs. Instead of reporting only costs (that can drastically change over time and by setting), resource use should be reported.

Considering health technology assessment of potential vaccines and monoclonal antibodies to reduce the burden of AMR, what are the most important resource use categories that should be reported in future AMR burden research?
Please rank the **5 most important** resource use categories

- Length of stay (by ward or specialty)
- Treatments
- Diagnostics
- Interventions
- Other hospital resources e.g. patient management
- Outpatient follow-up
- Primary care healthcare utilisation
- Absence from work
- Absence from care responsibilities
- Informal care provided / care-giver time
- Travel time
- Out of pocket expenses
- Other, please specify

Comment box:

Specific gaps

10. In systematic review 1 -focused on frequency measures- bloodstream, urinary tract, and respiratory tract infections were the most reported and analyzed. Which infectious syndromes should be prioritized for surveillance to be able to inform future AMR studies to support health technology assessment of potential vaccines and monoclonal antibodies to combat AMR?

Please **rank** the following infection types based on importance, refer to the gap mapping figure here(link) for more specific information.

(green – data available, red – few or no studies available)

- Bloodstream infections
- Urinary tract infections
- Surgical site infections
- Respiratory tract infections
- Skin and soft tissue infections
- Intraabdominal infections
- Others, such as bone and joint infections (free text)

11. Across two systematic reviews focused on burden of AMR (health and economic burden), bloodstream- and skin and soft tissue infections were most frequently analyzed, although the quality of studies was low. Which infectious syndromes should be prioritized in future AMR studies to support health technology assessment of potential vaccines and monoclonal antibodies to combat AMR?

Please **rank** the following infection types based on importance, refer to the gap mapping figure here(link) for more specific information.

(green – data available, red – few or no studies available)

- Bloodstream infections (AMR burden data available – low quality)
- Urinary tract infections
- Surgical site infections
- Respiratory tract infections
- Skin and soft tissue infections (AMR burden data available – low quality)
- Intraabdominal infections

Comment box:

If you don't feel you have the knowledge to answer this question, please tick this box and move to the next field. (checkbox)

1.1 Within bloodstream infections, please select the **3 most important** resistant pathogens:

1.1.1 Carbapenem resistant P. aeruginosa
1.1.2 Carbapenem resistant A. baumannii
1.1.3 3rd gen cephalosporin resistant E. coli (AMR burden data available)
1.1.4 Carbapenem resistant E. coli
1.1.5 3rd gen cephalosporin resistant K. pneumoniae
1.1.6 Carbapenem resistant K. pneumoniae
1.1.7 Methicillin resistant S. aureus (MRSA) (AMR burden data available)
1.1.8 Vancomycin resistant E. faecium (VRE) (AMR burden data available)

If you don't feel you have the knowledge to answer this question, please press next

Comment box:

1.2 Within urinary tract infections, please select the **3 most important** resistant pathogens:

1.2.1 Carbapenem resistant P. aeruginosa
1.2.2 Carbapenem resistant A. baumannii
1.2.3 3rd gen cephalosporin resistant E. coli (AMR burden data available)
1.2.4 Carbapenem resistant E. coli
1.2.5 3rd gen cephalosporin K. pneumoniae (AMR burden data available)
1.2.6 Carbapenem resistant K. pneumoniae
1.2.7 Vancomycin resistant E. faecium (VRE)

If you don't feel you have the knowledge to answer this question, please press next

Comment box:

1.3 Within surgical site infections, please select the **3 most important** resistant pathogens:

1.3.1 Carbapenem resistant P. aeruginosa
1.3.2 3rd gen cephalosporin resistant K. pneumoniae
1.3.3 Carbapenem resistant E. coli
1.3.4 3rd gen cephalosporin resistant E. coli
1.3.5 Carbapenem resistant K. pneumoniae
1.3.6 Methicillin resistant S. aureus (MRSA) (AMR burden data available)

If you don't feel you have the knowledge to answer this question, please press next

Comment box:

1.4 Within respiratory tract infections, please select the **3 most important** resistant pathogens:

1.4.1 Carbapenem resistant P. aeruginosa
1.4.2 Carbapenem resistant A. baumannii
1.4.3 3rd gen cephalosporin / carbapenem resistant E. coli
1.4.4 3rd gen cephalosporin / carbapenem resistant K. pneumoniae
1.4.5 Methicillin resistant S. aureus (MRSA) (AMR burden data available)

If you don't feel you have the knowledge to answer this question, please press next

Comment box:

1.5 Within intra-abdominal infections, please select the **3 most important** resistant pathogens:

1.5.1 Carbapenem resistant A. baumannii
1.5.2 3rd gen cephalosporin / carbapenem resistant E. coli
1.5.3 3rd gen cephalosporin / carbapenem resistant K. pneumoniae
1.5.4 Vancomycin resistant E. faecium (VRE)

If you don't feel you have the knowledge to answer this question, please press next

Comment box:

12. Mortality and excess length of stay associated with methicillin-resistant *S. aureus* bloodstream infections were frequently reported in studies conducted in Europe, while other health and economic outcomes, or risk-group specific estimates were rare. Overall, the included evidence was ranked as having a high risk of bias.

What research approach should be prioritized to fill the knowledge gaps on the AMR burden of methicillin-resistant *S. aureus* (MRSA) bloodstream infections, and to support health technology assessment of potential vaccines and monoclonal antibodies against this syndrome? (Please choose **one** approach)

- Conduct higher quality studies (low risk of bias) measuring the same outcome of MRSA BSI (mortality and excess length of stay)
- Conduct studies on health outcomes other than mortality and economic outcomes in terms of resource use associated with MRSA BSI (such as recurrence, organ failure, ICU admission, treatment, healthcare utilization)
- Conduct studies on the burden associated with MRSA BSIs in specific risk groups: i.e., immunocompromised, intravenous drug users, patients with malignancies.
- Other, please specify
- No opinion

Comment box:

13. The health burden of vancomycin resistant *Enterococcus* bloodstream infections was frequently reported in studies from high income countries (European and non-European). Overall, the included evidence on mortality was ranked as high risk of bias, and only one study assessed excess length of stay. Large heterogeneity was observed in terms of prevalence/ incidence of resistance in vancomycin resistant Enterococcus bloodstream isolates within Europe.

What research approach should be prioritized to fill the knowledge gaps for the AMR burden of vancomycin resistant *Enterococcus* bloodstream infections, to support health technology assessment of potential vaccines and monoclonal antibodies against this syndrome? (Please choose **one** approach)

- Conduct high quality studies (low risk of bias) on mortality and excess length of stay of vancomycin resistant *Enterococcus* bloodstream infections in Europe
- Conduct high quality studies on resistance prevalence of vancomycin resistant *Enterococcus* bloodstream infections in Europe
- Conduct studies on health outcomes other than mortality and resource use associated with vancomycin resistant *Enterococcus* bloodstream infections, such as recurrence, organ failure, ICU admission, treatment and healthcare utilisation
- Conduct studies on the burden of vancomycin resistant *Enterococcus* bloodstream infections in specific risk groups: i.e., immunocompromised and patients with malignancies
- Other, please specify
- No opinion

Comment box:

14. Mortality and excess length of stay of 3rd generation cephalosporin resistant *E. coli* bloodstream infections were frequently reported in studies conducted in Europe. The overall quality of evidence was low.

What research approach should be prioritized to fill the knowledge gaps for the AMR burden of 3rd generation cephalosporin resistant *E. coli* bloodstream infections to support health technology assessment of potential vaccines and monoclonal antibodies against this syndrome? (Please choose **one** approach)

- Conduct high quality studies on mortality and excess length of stay of 3rd generation cephalosporin resistant *E. coli* bloodstream infections
- Conduct studies on health outcomes other than mortality and resource use associated with 3rd generation cephalosporin resistant *E. coli* bloodstream infections, like recurrence, organ failure, ICU admission, treatment and healthcare utilisation
- Conduct studies on burden of 3rd generation cephalosporin resistant *E. coli* bloodstream infections in specific risk groups: i.e., immunocompromised, intravenous drug users, patients with malignancies.
- Other, please specify
- No opinion

Comment box:

15. Mortality and excess length of stay of methicillin-resistant *S. aureus* respiratory tract infections were frequently reported. Overall, the included evidence was ranked as high risk of bias. Large heterogeneity was observed in terms of prevalence/incidence of methicillin-resistant *S. aureus* respiratory tract infections.

What research approach should be prioritized to fill the knowledge gaps for the AMR burden of methicillin-resistant S. aureus respiratory tract infections, to support health technology assessment of potential vaccines and monoclonal antibodies against this syndrome? (Please choose one approach)

- Conduct high quality studies on mortality and excess length of stay of methicillin-resistant S. aureus respiratory tract infections in Europe

- Conduct high quality studies on resistance prevalence of methicillin-resistant S. aureus respiratory tract infections in Europe
- Conduct studies on health outcome other than mortality and resource use associated with methicillin-resistant S. aureus respiratory tract infections, like recurrence, organ failure, ICU admission, treatment and healthcare utilisation
- Conduct studies on the burden of methicillin-resistant S. aureus respiratory tract infections in specific risk groups: i.e., immunocompromised, or patients with malignancies or chronic lung disease
- Other, please specify
- No opinion

Comment box:

Please report any comments you have with regards to future research priorities for AMR burden studies (frequency measures, clinical and economic burden) related to assessment of the impact of vaccines and/or mono-clonal antibodies: *Free text field*

Data harmonization

The following questions address general recommendations for future study methodology and harmonization of definitions to generate more informative evidence on AMR burden to support health technology assessment of potential vaccines and monoclonal antibodies to reduce the burden of AMR.

16. When frequency measures (incidence/ prevalence) of resistant Enterobacterales infections are reported and when the sample size of the study is large enough, it should include disaggregated data stratified by pathogen (i.e. separate burden of E. coli, K. pneumonia and other pathogens)

Considering future health technology assessment of potential vaccines and monoclonal antibodies to reduce the burden of AMR, do you agree with this statement?

1=strongly disagree
2=moderately disagree
3=neither agree nor disagree
4=moderately agree
5= strongly agree
6= No expertise in this field

Comment box:

17. When AMR burden data (clinical and economic outcomes) is reported for resistant Enterobacterales infections and when the sample size of the study is large enough, it should include disaggregated data stratified by pathogen (separate burden of E. coli, K. pneumonia and other pathogens)

Considering future health technology assessment of potential vaccines and monoclonal antibodies to reduce the burden of AMR, do you agree with this statement?

1=strongly disagree
2=moderately disagree
3=neither agree nor disagree
4=moderately agree
5= strongly agree
6= No expertise in this field

Comment box:

18. Many studies on burden of AMR (clinical and economic burden) report on mortality outcomes, however, many different time-points are used, including, or excluding follow-up beyond hospital discharge. Harmonization of an all-cause mortality time point for studies assessing the burden of AMR is needed. Analysis of time varying confounding on outcomes is essential. What is the most appropriate time point for mortality assessment of drug-resistant infections that are treated in hospital?

Please rank based on importance and feasibility

in-hospital mortality
30-day mortality (after infection onset) <u>with</u> post discharge follow-up
30-day mortality (after infection onset) <u>without</u> post discharge follow-up
14-day mortality (after infection onset) <u>with</u> post discharge follow-up
14-day mortality (after infection onset) <u>without</u> post discharge follow-up
Other, please specify
No expertise in this field

Comment box:

19. Surveillance studies reporting drug resistance percentages should always report an estimation of the size of the population from which the study sample was taken, to allow for prevalence and incidence estimates generation.

Considering future health technology assessment of potential vaccines and monoclonal antibodies to reduce the burden of AMR, do you agree with this statement?

1=strongly disagree
2=moderately disagree
3=neither agree nor disagree
4=moderately agree
5= strongly agree
6= No expertise in this field

Comment box:

20. Studies assessing economic outcomes associated with AMR often lack information about characteristics of the included patient population, like frequency of comorbidities. Future studies should include this information to be better understand to which population the estimates apply and potential generalizability to other populations.

Considering future health technology assessment of potential vaccines and monoclonal antibodies to reduce the burden of AMR, do you agree with this statement?

1=strongly disagree
2=moderately disagree
3=neither agree nor disagree
4=moderately agree
5= strongly agree
6= No expertise in this field

Is there any additional important research agenda item(s) you would like to suggest considering future health technology assessment of potential vaccines and monoclonal antibodies to reduce the burden of AMR? Please specify.

Please report any comments you have with regards to data harmonization for AMR burden studies: *Free text field*

Please report any general comments related to this survey: *Free text field*

Round 2 – Second questionnaire in the Delphi study

Expert elicitation process: Round 2
In this round we will provide you with a list with the highest-ranking knowledge gaps, and all newly suggested statements by the expert panel. You are kindly asked to indicate the feasibility of filling each knowledge gap with future research. Please consider factors as the required study design, sample size, cost for conducting a study, setting and ease of implementation.
As a reminder, these knowledge gaps for human AMR burden data are based on three systematic reviews, focused on prevalence/incidence data (1), and associated clinical (2) and economic (3) outcomes from 1990-2023 (CRD 42022312795, CRD 2022322586, CRD 42022331400). Only pathogen-specific and infection-specific data were considered, e.*g.* outcomes associated with methicillin resistant *S. aureus* bloodstream infections. In this round, a shortened list is presented with the highest-ranking statements.
we will provide 25 statements indicating a specific or general research gap in human AMR burden research, for which we ask you to indicate the feasibility gap based on a Likert scale (1=strongly disagree, 2=moderately disagree, 3=neither agree nor disagree, 4=moderately agree, and 5= strongly agree, and an additional option: Don't know). For some statements, you will need to rank the elements based on feasibly. For some statements we seek consensus on the importance of the knowledge gap (as in the previous round). Please pay attention to the question text. Finally, there will be room for you to add comments

Herein, we invite you to participate in the second round. It includes <u>25</u> gap statements for your consideration.
An overview of the questionnaire content can be downloaded here (pdf)

General gaps

You will find X statements indicating a specific or general research gaps in human AMR burden research based on the systematic reviews, for which we ask you to indicate whether you agree with the proposed question based on a Likert scale (1=strongly disagree, 2=moderately disagree, 3=neither agree nor disagree, 4=moderately agree, and 5= strongly agree, and an additional option: Don't know). In some questions you will be asked to rank or select a maximal number of options.

1. **There is lack of data regarding the burden of AMR (clinical and economic impact) within pediatric populations**
   Consensus was reached on importance of this knowledge gap, please indicate the <u>feasibility of filling it with future research. Please consider ease of implementation factors as the required study design, sample size, costs, setting.</u>
   It is **<u>feasible</u>** to fill this knowledge gap.
   1=strongly disagree
   2=moderately disagree
   3=neither agree nor disagree
   4=moderately agree
   5= strongly agree
   6= Don't know

Comment box:

2. **There is lack of data on the burden of AMR (frequency, clinical and economic impact) from Eastern and Central European countries (Balkan, Poland, *etc*).**

   Consensus was reached on importance of this knowledge gap, please indicate the <u>feasibility of filling it with future research. Please consider factors as the required study design, sample size, costs, setting and ease of implementation</u>
   It is **<u>feasible</u>** to fill this knowledge gap.
   1=strongly disagree
   2=moderately disagree
   3=neither agree nor disagree
   4=moderately agree
   5= strongly agree
   6= Don't know

Comment box:

3. **There is lack of data on frequency measures of carbapenem resistant infections, caused by *P. aeruginosa, A. baumannii, E. coli and K. pneumoniae***

   This statement was revised as it did not reach the threshold required for agreement.

   a. This is **<u>an important knowledge gap</u>** for health technology assessment of potential vaccines and monoclonal antibodies to reduce the burden of AMR

      1=strongly disagree
      2=moderately disagree
      3=neither agree nor disagree
      4=moderately agree
      5= strongly agree
      6= No expertise in this field

   Comment box

   b. please indicate the <u>feasibility of filling it with future research. Please consider factors as the required study design, sample size, costs, setting and ease of implementation</u>

      It is **<u>feasible</u>** to fill this knowledge gap.
      1=strongly disagree
      2=moderately disagree
      3=neither agree nor disagree

4=moderately agree
5= strongly agree
6= Don't know

4. **There is lack of data on health and/or economic burden of AMR for carbapenem resistant infections, caused by *P. aeruginosa, A. baumannii, E. coli and K. pneumoniae***

This statement was revised as it did not reach the threshold required for agreement.

a. This is **an important knowledge gap** for health technology assessment of potential vaccines and monoclonal antibodies to reduce the burden of AMR

1=strongly disagree
2=moderately disagree
3=neither agree nor disagree
4=moderately agree
5= strongly agree
6= No expertise in this field

Comment box:

b. please indicate the feasibility of filling it with future research. Please consider factors as the required study design, sample size, costs, setting and ease of implementation

It is **feasible** to fill this knowledge gap.
1=strongly disagree
2=moderately disagree
3=neither agree nor disagree
4=moderately agree
5= strongly agree
6= Don't know

5. **Most AMR burden studies compare clinical and/or economic outcomes between patients with drug-resistant infections and patients with drug-susceptible infections (attributable burden), indicating the preventable burden of drug resistance. AMR burden studies comparing clinical and economic outcomes between patients with drug-resistant infections and patients without an infection (associated burden) are very rare, while this indicates the preventable burden of eliminating drug-resistant infections.**

Consensus was reached on importance of this knowledge gap, please indicate the feasibility of filling it with future research. Please consider factors as the required study design, sample size, costs, setting and ease of implementation

It is **feasible** to fill this knowledge gap.
1=strongly disagree
2=moderately disagree
3=neither agree nor disagree
4=moderately agree
5= strongly agree
6= No expertise in this field

Comment box:

6. **In AMR burden studies comparing clinical and/or economic outcomes between patients with drug-resistant infections to 2 comparator groups (drug-susceptible and patients without an infection), it is also important to compare the outcomes of patients with drug-susceptible infections to patients without an infection to estimate the burden of susceptible infections.**

a. This statement was added according to the suggestions in the previous round.

It is an **important knowledge gap** for health technology assessment of potential vaccines and monoclonal antibodies to reduce the burden of AMR

1=strongly disagree
2=moderately disagree
3=neither agree nor disagree
4=moderately agree
5= strongly agree
6= No expertise in this field

b. It is **feasible** to fill this knowledge gap.
1=strongly disagree
2=moderately disagree
3=neither agree nor disagree
4=moderately agree
5= strongly agree
6= Don't know

Comment box:

7. **For the clinical burden of AMR, most studies report mortality as an outcome; other outcomes are not frequently reported.**

In the previous round the most important outcomes per infection type were established. We now ask you to indicate the feasibility of filling it with future research. Please consider factors as the required study design, sample size, costs, setting and ease of implementation

Please choose the **8 most feasible and important research** domains:

BSI – Mortality
BSI – Clinical failure/ recurrence/relapse
BSI – Days in ICU following infection
UTI – Mortality
UTI – Clinical failure/ recurrence/relapse
UTI – Physical debilitation/deconditioning (temporary and/or permanent)
RTI – Mortality
RTI – Clinical failure/ recurrence/relapse
RTI – Days in ICU following infection
RTI – Physical debilitation/deconditioning (temporary and/or permanent)
SSI – Mortality
SSI – Clinical failure/ recurrence/relapse
SSI – Days in ICU following infection

Comment box:

8. It would be important to report frequency measures (*i.e. prevalence, incidence*) of infection for the following risk groups – as defined in the previous round, by order of importance:

please rank the following according to feasibility of filling it with future research. Please consider factors as the required study design, sample size, costs, setting and ease of implementation

- Elderly
- Neonates
- Surgical patients
- Children
- Transplanted patients
- Patients with hemato-oncological malignancies

Comment box:

9. It would be important to report on AMR burden (health and economic outcomes) for the following risk groups – as defined in the previous round, by order of importance:

please rank the following according to feasibility of filling it with future research. Please consider factors as the required study design, sample size, costs, setting and ease of implementation

1) Elderly
2) Neonates
3) Surgical patients
4) Immunocompromised patients
5) Patients with hemato-oncological malignancies

Comment box:

10. Very few studies report on the economic outcomes associated with AMR, and those that do tend to report crude costs (cost in $) rather than resource use (i.e. 1 CT scan, 1 course of antibiotics). Clinical studies on the burden of AMR should include estimates of resource use associated with infection for a minimal set of items that can then be linked to unit costs.

Consensus was reached on importance of this knowledge gap, please indicate the feasibility of filling it with future research. Please consider factors as the required study design, sample size, costs, setting and ease of implementation
It is **feasible** to fill this knowledge gap.

1=strongly disagree
2=moderately disagree
3=neither agree nor disagree
4=moderately agree
5= strongly agree
6= Don't know

Comment box:

11. Important economic outcomes associated with drug-resistant infections were selected in the previous round.
please rank the following according to feasibility of filling it with future research. Please consider factors as the required study design, sample size, costs, setting and ease of implementation

- Treatments
- Length of stay (by ward or specialty)
- Interventions
- Absence from work
- Diagnostics

Comment box:

Specific gaps

12. **In the previous round the most important knowledge gaps on specific infection types for generating research on frequency measures were established – ordered by importance.**
We now ask you to indicate the feasibility of filling it with future research. Please consider factors as the required study design, sample size, costs, setting and ease of implementation

    1. Bloodstream infections
    2. Urinary tract infections
    3. Respiratory tract infections
    4. Surgical site infections

13. **In the previous round the most important knowledge gaps on specific infection types for generating research on AMR economic and health burden were established**

    We now ask you to indicate the feasibility of filling it with future research. Please consider factors as the required study design, sample size, costs, setting and ease of implementation
Please choose the **6 most important and feasible** research domains from the list below:

    BSI – 3rd gen cephalosporin resistant K. pneumoniae
    BSI – Carbapenem resistant K. pneumoniae
    BSI – Carbapenem resistant A. baumannii
    UTI – 3rd gen cephalosporin resistant E. coli
    UTI – Carbapenem resistant E. coli
    UTI – 3rd gen cephalosporin K. pneumoniae
    SSI – Methicillin resistant S. aureus (MRSA)
    SSI – 3rd gen cephalosporin resistant K. pneumoniae
    SSI – 3rd gen cephalosporin resistant E. coli
    RTI – Carbapenem resistant P. aeruginosa
    RTI – Carbapenem resistant K. pneumoniae
    RTI – Carbapenem resistant A. baumannii

14. **Consensus was reached on the need for future higher quality studies (low risk of bias) measuring the mortality and length of hospital stay of patients with MRSA BSI**

    please indicate the feasibility of filling it with future research. Please consider factors as the required study design, sample size, costs, setting and ease of implementation
It is **feasible** to fill this knowledge gap.

    1=strongly disagree
    2=moderately disagree
    3=neither agree nor disagree
    4=moderately agree
    5= strongly agree
    6= Don't know

15. **Consensus was reached on the need for future studies on health outcomes other than mortality and economic outcomes in terms of resource use associated with MRSA BSI (such as recurrence, organ failure, ICU admission, treatment, healthcare utilization)**

    please indicate the feasibility of filling it with future research. Please consider factors as the required study design, sample size, costs, setting and ease of implementation
It is **feasible** to fill this knowledge gap.

    1=strongly disagree
    2=moderately disagree
    3=neither agree nor disagree
    4=moderately agree
    5= strongly agree
    6= Don't know

16. **Consensus was reached on the need for higher quality future studies measuring mortality and excess length of stay of vancomycin resistant Enterococcus bloodstream infections in Europe**

    please indicate the feasibility of filling it with future research. Please consider factors as the required study design, sample size, costs, setting and ease of implementation
It is **feasible** to fill this knowledge gap.

    1=strongly disagree
    2=moderately disagree
    3=neither agree nor disagree
    4=moderately agree
    5= strongly agree

6= Don't know

Comment box:

17. Consensus was reached on the need for higher quality studies on mortality and excess length of stay of 3rd generation cephalosporin resistant E. coli bloodstream infections

please indicate the feasibility of filling it with future research. Please consider factors as the required study design, sample size, costs, setting and ease of implementation

It is **feasible** to fill this knowledge gap.

1=strongly disagree
2=moderately disagree
3=neither agree nor disagree
4=moderately agree
5= strongly agree
6= Don't know

Comment box:

18. Consensus was reached on the need for future studies on health outcomes other than mortality and resource use associated with 3rd generation cephalosporin resistant E. coli bloodstream infections, like recurrence, organ failure, ICU admission, treatment and healthcare utilization

please indicate the feasibility of filling it with future research. Please consider factors as the required study design, sample size, costs, setting and ease of implementation

It is **feasible** to fill this knowledge gap.

1=strongly disagree
2=moderately disagree
3=neither agree nor disagree
4=moderately agree
5= strongly agree
6= Don't know

Comment box:

19. Consensus was reached on the need for high quality studies on mortality and excess length of stay of methicillin-resistant S. aureus respiratory tract infections in Europe

please indicate the feasibility of filling it with future research. Please consider factors as the required study design, sample size, costs, setting and ease of implementation

It is **feasible** to fill this knowledge gap.

1=strongly disagree
2=moderately disagree
3=neither agree nor disagree
4=moderately agree
5= strongly agree
6= Don't know

Comment box:

Please report any comments you have with regards to feasibility of future research: *Free text field*

Data harmonization

The following questions address general recommendations for future study methodology and harmonization of definitions to generate more informative evidence on AMR burden to support health technology assessment of potential vaccines and monoclonal antibodies to reduce the burden of AMR.

20. Consensus was reached on the recommendation that when frequency measures (incidence/ prevalence) of resistant Enterobacterales infections are reported and when the sample size of the study is large enough, disaggregated data stratified by pathogen (i.e. separate burden of E. coli, K. pneumonia and other pathogens) should be reported.

please indicate the feasibility of filling it with future research. Please consider factors as the required study design, sample size, costs, setting and ease of implementation

It is **feasible** to fill this knowledge gap.

1=strongly disagree
2=moderately disagree
3=neither agree nor disagree
4=moderately agree
5= strongly agree
6= Don't know

Comment box:

21. Consensus was reached on the recommendation that when AMR burden data (clinical and economic outcomes) is reported for resistant Enterobacterales infections and when the sample size of the study is large enough, disaggregated data stratified by pathogen (separate burden of E. coli, K. pneumonia and other pathogens) should be reported.

please indicate the feasibility of filling it with future research. Please consider factors as the required study design, sample size, costs, setting and ease of implementation
It is **feasible** to fill this knowledge gap.

1=strongly disagree
2=moderately disagree
3=neither agree nor disagree
4=moderately agree
5= strongly agree
6= Don’t know

Comment box:

22. **Many studies on burden of AMR (clinical and economic burden) report on mortality outcomes, however, many different time-points are used, including, or excluding follow-up beyond hospital discharge. Harmonization of an all-cause mortality time point for studies assessing the burden of AMR is needed. Analysis of time varying confounding on outcomes is essential. What is the most important time point for mortality assessment of drug-resistant infections that are treated in hospital?**

Please rank based on importance only!

in-hospital mortality
30-day mortality (after infection onset) with post discharge follow-up
30-day mortality (after infection onset) without post discharge follow-up
14-day mortality (after infection onset) with post discharge follow-up
14-day mortality (after infection onset) without post discharge follow-up
Other, please specify
No expertise in this field

Comment box:

23. **Many studies on burden of AMR (clinical and economic burden) report on mortality outcomes, however, many different time-points are used, including, or excluding follow-up beyond hospital discharge. Harmonization of an all-cause mortality time point for studies assessing the burden of AMR is needed. Analysis of time varying confounding on outcomes is essential. What is the most feasible time point for mortality assessment of drug-resistant infections that are treated in hospital?**

Please rank based on feasibility only!

in-hospital mortality
30-day mortality (after infection onset) with post discharge follow-up
30-day mortality (after infection onset) without post discharge follow-up
14-day mortality (after infection onset) with post discharge follow-up
14-day mortality (after infection onset) without post discharge follow-up
Other, please specify
No expertise in this field

Comment box:

24. **Consensus was reached on the recommendation that surveillance studies reporting drug resistance percentages should always report an estimation of the size of the population from which the study sample was taken, to allow for prevalence and incidence estimates generation.**
please indicate the feasibility of filling it with future research. Please consider factors as the required study design, sample size, costs, setting and ease of implementation
It is **feasible** to fill this knowledge gap.

1=strongly disagree
2=moderately disagree
3=neither agree nor disagree
4=moderately agree
5= strongly agree
6= Don’t know

Comment box: Please comment on which measures would be worth reporting:

25. **Consensus was reached on the recommendation that studies assessing economic outcomes associated with AMR should report information on the characteristics of the included patient population, like frequency of comorbidities to be better understand to which population the estimates apply and potential generalizability to other populations.**
please indicate the feasibility of filling it with future research. Please consider factors as the required study design, sample size, costs, setting and ease of implementation
It is **feasible** to fill this knowledge gap.

1=strongly disagree
2=moderately disagree
3=neither agree nor disagree
4=moderately agree
5= strongly agree
6= Don’t know

Comment box:

Is there any additional important research agenda item(s) you would like to suggest considering future health technology assessment of potential vaccines and monoclonal antibodies to reduce the burden of AMR? Please specify.

Please report any comments you have with regards to data harmonization for AMR burden studies: *Free text field*

Please report any general comments related to this survey: *Free text field*
